# Economic Impact of Changes in Neonatal Intensive Care Unit Ventilation Strategies with the Advent of New Noninvasive Ventilation Techniques: A Review and Proposed Assessment Framework for High Flow Therapy as a Routine Respiratory Support Paradigm

**DOI:** 10.36469/9840

**Published:** 2015-08-19

**Authors:** Jan B. Pietzsch, Abigail M. Garner, Michael McQueen

**Affiliations:** 1 Wing Tech Inc., Menlo Park, CA USA; 2 Division of Neonatology, Banner Estrella, Thunderbird, and Del E. Webb Medical Centers, Phoenix, AZ, USA

**Keywords:** cost, economics, hft, ncpap, cmv, respiratory support, neonatal care

## Abstract

**Background:** High flow therapy (HFT) has been demonstrated to be a safe and effective noninvasive respiratory support technique for the treatment of pre-term infants in neonatal intensive care.

**Objectives:** Our objective was to develop a quantitative framework based on available evidence to estimate the economic impact of adoption of a HFT respiratory support strategy compared to current standard of care.

**Methods:** Model parameters were derived from a recent study comparing respiratory modality utilization between five US-based neonatal intensive care units (NICUs) adopting a HFT strategy and a larger pool of NICUs in the Vermont-Oxford Network (VON), and from single center experience. We computed the total cost difference between the respiratory support strategies based on published cost data. Parameter uncertainty was tested in sensitivity analyses.

**Results:** The constructed model projected expected cost savings of $2,317 for the HFT strategy for the base case. Results were sensitive to length of HFT use, length of CMV, cost of HFT, and length of nCPAP support.

**Conclusions:** Adoption of a HFT strategy appears to be associated with meaningful savings in total NICU episode of care costs, primarily because of reductions in the time of conventional mechanical ventilation. Further research is warranted to substantiate these findings.

## INTRODUCTION

In the last decades, advances in neonatal intensive care have greatly contributed to improvements in survival and outcomes of preterm infants. Management of respiratory failure, which remains a key clinical challenge in treating pre-term infants, has benefited from advances in respiratory support techniques that focus on reducing the exposure to invasive mechanical ventilation to further reduce lung injury and improve longterm outcomes.[1,2] The increased use of nasal continuous positive airway pressure (nCPAP), and more recently, of high flow nasal cannula therapy (HFT) as an early noninvasive respiratory support mode provide opportunities for further improvements in respiratory outcomes. At the same time, use of less invasive respiratory support technologies might provide an opportunity to contribute to reductions in the total costs of care in a neonatal intensive care unit (NICU), which continue to be staggering and remain a concern to caregivers, hospital administrators and payers.

While a number of studies have investigated the safety and clinical effectiveness of HFT compared to nCPAP, and found HFT to be associated with comparable outcomes to nCPAP,[3,4,5] only limited work has been done to understand the economic impact of adopting a HFT-based respiratory support strategy, compared to other strategies. In this article, we propose a quantitative framework for the economic evaluation of the adoption of a HFT strategy. This model is based on recent data comparing utilization data associated with adoption of a HFT compared to non-HFT strategy, and uses information and cost from the published literature to complement these data. The outcome of interest is the difference in total cost per episode of care for newborns of average twenty eight weeks of gestational age, resembling a typical very low birth weight (VLBW) cohort. The analysis is performed from the perspective of a NICU in the United States healthcare system setting.

## METHODS

### Clinical Utilization Data

In a structured search, two recent data sets were identified that investigated differences in respiratory support utilization and other relevant utilization parameters in patients treated in NICUs that have adopted HFT as a routine support paradigm ("HFT strategy"), versus patients treated in a NICU setting where HFT is not a routine support paradigm ("non-HFT strategy"). The first was a study investigating differences between five US-based centers that have adopted HFT as a routine support paradigm, and data reported by the Vermont Oxford Network (VON).[6] The second was data from a case series collected at a level III NICU (Banner Thunderbird Medical Center, Glendale, AZ). This operational case study investigated utilization rates for the year 2011, with the NICU relying for the first two quarters (Q1 and Q2) on a conventional respiratory support approach, and the remaining quarters (Q3 and Q4) adopting HFT as a routine support paradigm. A PubMed search, performed in October 2013, did not identify any additional published studies reporting HFT utilization data.

The data from the five HFT centers reported in the first study6 [r]epresent combined very low birth weight (VLBW) admissions of 446 in 2009, 429 in 2010 and 488 in 2011, totaling 1,363 VLBW infants. The VON database the authors used for comparison consisted 58,661 VLBW admissions in 2009, 57,992 in 2010 and 59,946 in 2011, totaling 176,599 VLBW babies.

The second data set, of the single NICU,[7] which was used to complement the VON data, consisted of 8,973 reported NICU days (4,288 in Q1-Q2/2011 pre-, and 4,688 in Q3-Q4/2011 post-introduction of HFT routine support). This unit is a Level IIIB AAP Category NICU, with approximately 450 admissions per year. For the data of Q1-Q2 2011, approximately 5% of admissions were <28 weeks gestation; for Q3-Q4, the percentage of infants admitted <28 weeks was approximately 10%.

For construction of the model, parameters of the HFT center study6 were included if the VON mean values, taken to represent the population, were outside the 95% confidence intervals of the values reported for the respective parameters for the five HFT centers. Relevant parameters meeting this criterion were percent utilization of continuous mechanical ventilation (considering both endotracheal and nasal conventional mechanical ventilation [CMV]), percent utilization of nCPAP, percent utilization of HFT/HFNC, percent surfactant use, and total length of stay (LOS). From the second, smaller data set (single NICU), only two additional parameters were included that were deemed significant based on the collected data: utilization of blood gases and of chest x-rays. These data points were not collected in the larger study.[6]

### Framing of Economic Evaluation

The framework for economic evaluation was developed to facilitate estimation of the differences in total cost per NICU episode of care, comparing the two respiratory support strategies (HFT routine support vs. non-HFT routine support). The total cost difference (TCD) was defined as the sum of individual savings or added costs stemming from utilization differences multiplied with the respective unit cost, and is described in the formula below. All cost inputs were adjusted to reflect 2013 U.S. costs.

$$TCD = (CMV_{HFT} - CMV_{non\text{-}HFT})*C_{CMV} + (nCPAP_{HFT} - nCPAP_{non\text{-}HFT})*C_{nCPAP} + (HFT_{HFT} - HFT_{non\text{-}HFT})*C_{HFT} + (Sfct._{HFT} - Sfct._{non\text{-}HFT})*C_{Sfct.} + (LOS_{HFT} - LOS_{non\text{-}HFT})*C_{NICU\ day} + (BG_{HFT} - BG_{non\text{-}HFT})*C_{BG} + (CXR_{HFT} - CXR_{non\text{-}HFT})*C_{CXR}$$

Legend: TCD: Total cost difference; CMV: utilization of conventional mechanical ventilation; nCPAP: nasal CPAP; HFT: utilization of high flow therapy (HFNC for non-HFT strategy); Sfct.: Utilization of surfactant; LOS: Length of stay (in days); BG: Utilization of blood gases (number per total NICU episode); CXR: chest x-ray (number per total NICU stay); C: unit cost (of index).

### Utilization and Cost Parameters

The primary utilization data obtained from the two identified studies are listed in Table 1. As only percentages for use of respiratory support modalities were reported in these studies, but no actual length of ventilation given for the respective respiratory support modality, we estimated length of ventilation based on a recently published study comparing new respiratory support strategies to conventional strategy.[8] In addition, further assumptions were made using the reported total length of ventilation for newborns born at 28 weeks gestational age.[9,10] Because of the limited evidence available for duration of ventilation, effects of changes in these parameters were comprehensively studied in sensitivity analyses.

Cost estimates were obtained from the published literature and publicly available records, and were converted to 2013 costs where needed. Respiratory-support dependent per-diem costs of NICU care were based on a prior publication[11] that reported costs derived from daily charges for patients in a tertiary care NICU. These daily charges considered all charges for patient care, including non-personnel and personnel charges, as well as allocated values for overhead expenses. Costs were derived in this publication by using cost center-specific cost-to-charge ratios.[11] Cost of surfactant was based on current cost of Survanta SDV.

Cost of one NICU day (at end of treatment episode) was estimated based on per-diem cost reported for infants on standard nasal cannula.[11] Cost of a blood gas test and cost of a chest x-ray exam were obtained from current charges published for a representative NICU, and converted to cost using the current region-specific Medicare cost-to-charge ratio. All cost inputs are listed in Table 2.

### Base Case and Sensitivity Analyses

The base case computation of total cost difference used the parameter inputs are shown in Tables 1 and 2. Uncertainty in parameter values was assessed by performing one-way sensitivity analyses. All cost inputs were varied by +/- 25%, in line with standard practice in health-economic analyses. In absence of further data describing parameter uncertainty, all inputs for length of ventilation, by strategy, were also varied conservatively by +/- 25%. In addition, several threshold analyses were performed.

**Table 1.** Model Assumptions: Utilization Parameters and Estimated Values for HFT and Non-HFT Strategy

| Study-derived Utilization Parameters | Non-HFT Strategy | HFT Strategy | Source |
|---|---|---|---|
| CMV use | 64.6% | 58.5% | (13) |
| nCPAP use | 68.6% | 30.3% | (13) |
| HFT use (HFNC in case of non-HFT strategy) | 53.0% | 73.3% | (13) |
| Surfactant use | 62.4% | 59.1% | (13) |
| Total length of stay | 60.7 | 62.0 | (13) |
| Blood gases (per patient, per total NICU stay) | 18.5 | 9.0 | Estimate based on (7) |
| Chest X-rays (per patient, per total NICU stay) | 9.6 | 4.2 | Estimate based on (7) |
| **Estimated length of use of respiratory support mode (if used)** | **Non-HFT Strategy** | **HFT Strategy** | **Source** |
| Length of CMV use (days) | 16.2 | 9.1 | Estimate based on (8), supported by (10) |
| Length of nCPAP use (days) | 13.0 | 9.5 | Estimate based on (8) |
| Length of HFT/HFNC use (days) | 11.5 | 23.0 | Estimate based on singlecenter data (HFT strategy) and assumption for non HFT-strategy) |

HFT: high flow therapy; CMV: conventional mechanical ventilitation; nCPAP: Nasal continuous positive airway pressure; HFNC: high flow nasal cannula

**Table 2.** Overview of Cost Inputs

| Cost Parameters | Base Case | Sensitivity Analysis Range | Source |
|---|---|---|---|
| Cost per NICU day, conventional mechanical ventilation (CMV) | $2232 | $1674-2790 | (11), inflated to 2013 using CPI |
| Cost per NICU day, nCPAP ventilation | $1904 | $1428-2380 | (11), inflated to 2013 using CPI |
| Cost per NICU day, high flow therapy (HFT) | $1904 | $1428-2380 | (11) (considered same as nCPAP), inflated to 2013 using CPI |
| Cost of surfactant | $982.37 | $737-1228 | Price of 8ml of Survanta SDV(25mg/ml) |
| Cost of one NICU day at end of treatment episode | $860 | $645-1075 | Cost per NICU day of standard nasal cannula reported in (11), inflated to 2013 using CPI |
| Cost of single blood gas test | $32.48 | $23-41 | Based on charge from (14), adjusted using FY 2013 Medicare cost-to-charge ratio of 0.295 |
| Cost of single chest X-ray | $112.91 | $85-141 | Based on charge from (14), adjusted using FY 2013 Medicare cost-to-charge ratio of 0.295 |

NICU: neonatal intensive care unit; CPI: consumer price index; nCPAP: Nasal continuous positive airway pressure

## RESULTS

The base case assumptions about utilization and length of use of the various respiratory modalities lead to a total average length of ventilation (considered to include CMV, nCPAP, HFT) of 25.48 days (8.92 days nCPAP, 6.10 days HFT, 10.47 days CMV) for the non-HFT strategy cohort and 25.06 days (2.88 days nCPAP, 16.86 days HFT, 5.32 days CMV) for the HFT strategy cohort. This total duration seems realistic given the 25/75-percentiles of 8 days/30 days reported by Wilson et al.9 for gestational age of 28 weeks, which corresponds with the average gestational age of the two studies from which utilization parameters where drawn.

The base case projection of cost difference for the total episode of care showed savings of $2,317 when adopting the HFT vs. non-HFT strategy. The differences in utilization rates of the three different respiratory support strategies lead to substantial shifts in the individual cost contributions by modality (Table 3). As expected, the lower utilization of CMV in the HFT strategy reduces the respective cost component by more than half. At the same time, the HFT-associated cost component increases almost threefold, and the nCPAP-related costs are reduced to a third of the non-HFT strategy cost. Considering these three cost components only, a difference in total episode-of-care costs of $2,481 resulted, in favor of HFT-strategy. Cost differences from differences in usage of blood gas tests, chest X-rays, and surfactant use result in an additional estimated saving of $954 associated with the HFT vs. non-HFT strategy. The observed increase in length of stay of 1.3 days observed with the HFT strategy result in additional cost for HFT of $1,118. Altogether, these differences in cost add up to the reported saving of $2,317.

The results of the sensitivity analyses are shown in Table 4 and Figure 1 (Tornado Diagram). When varying each of the input parameters by +/-25% from baseline, length of HFT (HFT-strategy), length of CMV

(non-HFT strategy), cost of HFT per day, and length of CPAP use for the non-HFT strategy had the highest impact on the projected results. If HFT was used for shorter period of time than the base case assumption of 23 days, savings for HFT increased. Conversely, if duration of CMV for the non-HFT strategy increased from the base duration of 16.2 days, HFT strategy savings increased. Increased HFT therapy cost lead to lower amount of savings for this strategy. A longer usage of nCPAP for the non-HFT strategy also led to increased savings of the HFT strategy. Variations in the remainder of parameters did not materially affect the expected outcome of a total saving for the HFT strategy. Additional threshold analyses showed that HFT strategy was cost-saving for up to 24.6 days of use, all other parameters left unchanged.

**Table 3. Cost Difference between HFT and non-HFT Strategy**

| | CPAP Use | | HFT Use | | CMV Use | | Surfactant Use | Blood Gases | Chest X-Rays | Length of Stay (Days) | |
|---|---|---|---|---|---|---|---|---|---|---|---|
| | Percent Usage | Estimated avg. duration (days) of CPAP if used | Percent Usage | Estimated avg. duration of HFT if used | Percent Usage | Estimated avg. duration of CMV if used | | | | | |
| Non-HFT Strategy | 68.6% | 13.0 | 53.0% | 11.5 | 64.6% | 16.2 | 62.40% | 18.5 | 9.6 | 60.70 | |
| HFT Strategy | 30.3% | 9.5 | 73.3% | 23.0 | 58.5% | 9.1 | 59.10% | 9.0 | 4.2 | 62.00 | |
| Absolute diff. HFT vs non-HFT | -38.30% | (3.5) | 20.30% | 11.5 | -6.10% | (7.1) | -3.30% | (9.54) | (5.42) | 1.30 | Aggregate total cost difference HFT vs non-HFT Strategy |
| Resulting cost non-HFT strategy | $16 980 | | $11 605 | | $23 358 | | $613 | $601 | $1081 | | |
| Resulting cost HFT strategy | $5481 | | $32 100 | | $11 822 | | $581 | $291 | $469 | | |
| Resulting cost difference HFT vs non-HFT | $(11 499) | | $20 495 | | $(11 476) | | $(32) | $(310) | $(612) | $1118 | $(2317) |

Numbers in parenthesis reflect cost savings associated with the HFT strategy compared to non-HFT.

**Table 4.** Input Values and Results of One-way Sensitivity Analyses

| | Sensitivity analysis inputs | | | Results | | Span |
|---|---|---|---|---|---|---|
| | Base Case | -25% | 25% | -25% | 25% | |
| Length of HFT (HFT) | 23.0 | 17.3 | 28.8 | $(10 342) | $5708 | $16 050 |
| Length of CMV (non-HFT) | 16.2 | 12.2 | 20.3 | $3523 | $(8157) | $11 680 |
| Cost of HFT day | $1904 | $1428 | $2380 | $(7441) | $2807 | $10 248 |
| Length of nCPAP vent (non-HFT) | 13.0 | 9.8 | 16.3 | $1928 | $(6562) | $8490 |
| Length of CMV (HFT) | 9.1 | 6.8 | 11.4 | $(5287) | $654 | $5941 |
| Length of HFT (non-HFT) | 11.5 | 8.6 | 14.4 | $584 | $(5218) | $5802 |
| Cost of nCPAP day | $1904 | $1428 | $2380 | $558 | $(5192) | $5750 |
| Cost of CMV day | $2232 | $1674 | $2790 | $552 | $(5186) | $5738 |
| Length of nCPAP vent (HFT) | 9.5 | 7.1 | 11.9 | $(3687) | $(947) | $2740 |
| Cost of NICU day at end of episode | $860 | $645 | $1075 | $(2596) | $(2037) | $559 |
| Cost of chest X-ray | $113 | $85 | $141 | $(2164) | $(2470) | $306 |
| Cost of blood gas chest | $32 | $24 | $41 | $(2239) | $(2394) | $155 |
| Cost of surfactant | $982 | $737 | $1228 | $(2309) | $(2325) | $16 |

**Figure 1.** Tornado Diagram showing Results of One-way Sensitivity Analyses (all parameters varied by +/- 25%)

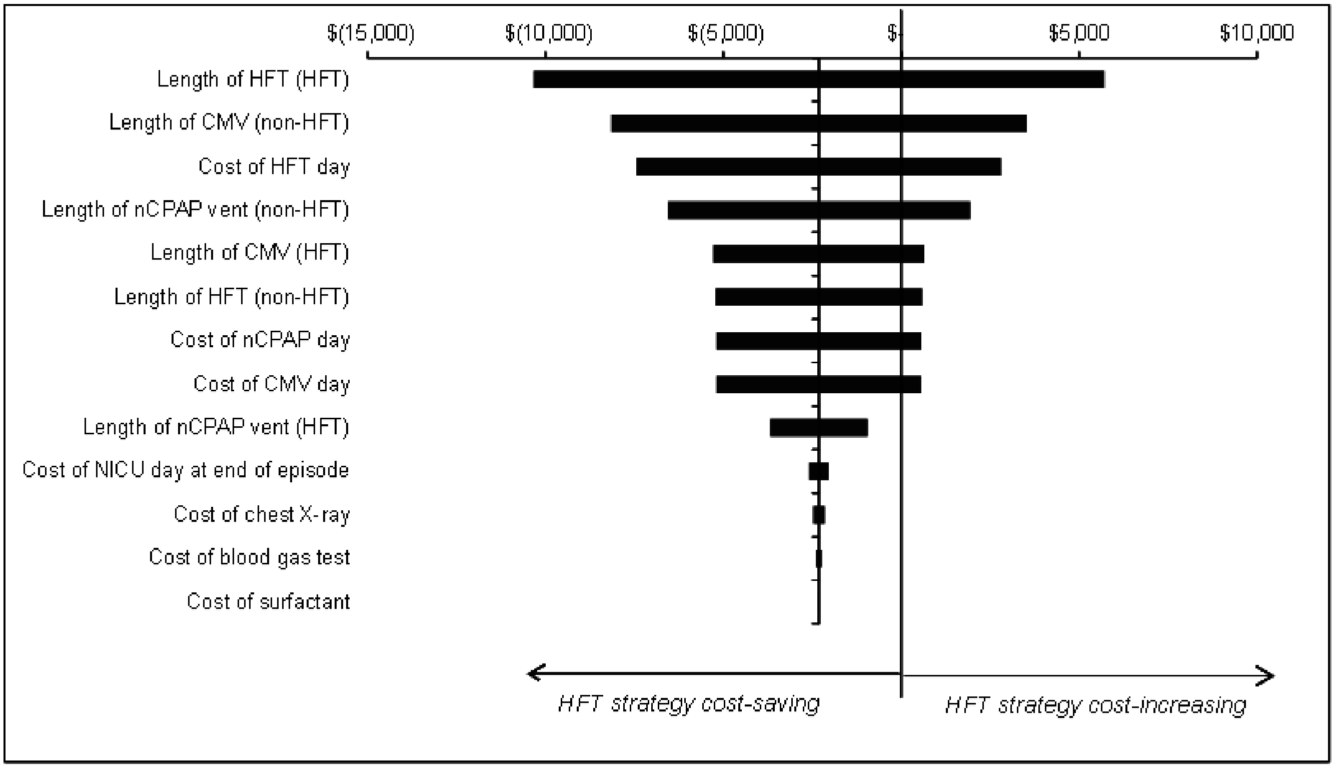

## DISCUSSION

While increasing evidence exists about comparable clinical efficacy of HFT and nCPAP as respiratory support strategies for preterm infants,[3,4,5,12] limited information exists about the potential economic implications of adopting less invasive respiratory support strategies, specifically HFT. However, this type of information is necessary in addition to clinical evidence to inform decision-making by providers and payers about the potential adoption of new treatment paradigms.

This study presents a framework for the estimation of total cost differences between a strategy that relies on HFT as a routine respiratory support paradigm, and a more conventional paradigm relying primarily on CMV and nCPAP. Its purpose, beyond presentation of initial results based on the current evidence about strategy-dependent modality utilization rates and lengths of use, is to present a framework that provides input for future prospective data collection and analyses.

The obtained initial results suggest that adoption of a HFT strategy may be associated with potential cost savings of 3-5% of total NICU episode of care costs, when considering total costs in a typical range of $50,000-$75,000. As would be expected, a major driver of this cost-reduction is the reduction of utilization and length of use of continuous mechanical ventilation. Potential cost savings also depend substantially on the length of HFT use. Specifically, as the sensitivity analyses show, in NICUs that are more accustomed to prolonged ventilator management, cost savings could reach savings in the range of $5,000 to $8,000 per case, or even higher. The HFT-associated reduction in ancillary procedures, such as chest x-rays and blood gases, while economically a less pronounced factor, is an important clinical factor.

Our analysis is subject to several limitations. First, the validity of the presented framework depends on the inclusion of the appropriate utilization parameters, the quality of the evidence informing the specific realizations of these parameters, and the use of appropriate cost inputs. Our choice of included parameters was based on statistically meaningful differences observed for these parameters between the data of the five HFT centers and the data from the larger VON network. In addition, blood gases and chest x-rays were included as additional parameters because the one center-experience showed statistically significant differences in these parameters pre- and post-introduction of a HFT strategy. Percent utilization of the various ventilation/respiratory support paradigms obtained from the five HFT centers (total of 1,363 VLBW newborns) and the VON network (58,661 VLBW newborns) was drawn from a reasonably large sample of patients. However, length of utilization, given a specific modality was used, was not collected in these studies. We therefore needed to rely on estimates from a recent study comparing a previous standard-of-care paradigm to a new paradigm relying more heavily on non-invasive ventilation.8 This study only reported the experience of sixty newborns for the old strategy, and sixty one newborns for the new strategy. Second, the two strategies in this study likely did not completely resemble the strategies that inform our percent utilization rates. Third, our analysis needed to rely on analogy to nCPAP use and on expert opinion to estimate length of use of HFT for the HFT and non-HFT strategies. However, this occurred in conjunction with validation of the projected total average ventilation time across all modalities, which at approximately 25 days for both strategies seems reasonable in light of reported length of ventilation data for newborns of average gestational age of 28 weeks.[9]

The estimates of per-diem NICU costs, stratified by type of ventilation/respiratory support, reported in a prior study[11] provide appropriate cost input to derive total cost differences that result from changes in utilization of the different respiratory support modalities. The study used is one of very few, if not the only one, that has estimated per-diem costs based on a rigorous review of charges in a tertiary care NICU, and subsequent conversion to costs using cost-to-charge ratios. However, that study assumed HFT per-diem cost to be similar to n-CPAP per-diem cost. While we worked with the same assumption in the absence of other information, we believe further prospective study is warranted to investigate the validity of this assumption.

Among the reasons that would suggest a potentially lower cost of HFT than nCPAP is the lower technological and clinical complexity of HFT, and the lower price point of the technology. Of note, the per-diem NICU cost estimate assumed for patients on nCPAP or HFT is $1,904, while the published study assumes only $860 (in 2013 cost) per-diem on nasal cannula therapy. It could reasonably be argued that the true HFT costs lie somewhere between this value and the nCPAP cost estimate of $1,904. As such, our analysis and the potential cost savings projected for HFT may be considered conservative. This is further underscored by the fact that reductions in required staff time are not considered in this analysis. Finally, we note that cost and potential clinical differences exist between high flow nasal cannula (HFNC) and HFT therapies.

In summary, our analysis suggests that adoption of a HFT strategy can be expected to lead to changes in utilization rates of respiratory support modalities that lead to measured overall savings in total NICU cost of care. Information from the presented framework and the sensitivity analyses provides insight into parameters that should in particular be studied in future clinical studies to improve the current evidence base that is still limited.

## Conflict of Interest Declaration

The views expressed in the submitted article are the authors' and not an official position of an institution or funder.

Wing Tech Inc. (JBP, AMG) provided consulting services for Vapotherm in developing the underlying health-economic model.
